# All-Inside ACL Reconstruction: Short Femoral and Tibial Socket Technique

**DOI:** 10.1177/26350254231212232

**Published:** 2024-04-02

**Authors:** Dai Sato, Samuel G. Moulton, Drew A. Lansdown, C. Benjamin Ma

**Affiliations:** * Department of Orthopaedic Surgery, Sports Medicine and Shoulder Surgery, University of California, San Francisco, San Francisco, California, USA; † Department of Orthopedic Surgery, Faculty of Medicine, Graduate School of Medicine, Hokkaido University, Kita-ku, Japan; Investigation performed at Department of Orthopaedic Surgery, Sports Medicine and Shoulder Surgery, University of California, San Francisco, San Francisco, California, USA

**Keywords:** arthroscopy, ACL, all-inside ACL reconstruction, short socket, graft mature

## Abstract

**Background:**

Recently, there has been an increase in interest in all-inside anterior cruciate ligament (ACL) reconstruction (ACLR). Regarding graft maturity, graft contact with the bone tunnel is important. All-inside ACLR with short femoral and tibial sockets might allow to increase the contact between the graft and the socket (ie, no interference screw and contact between the graft and bottom of the socket) and can improve graft maturing.

**Indications:**

All-inside ACLR with short sockets technique is indicated in patients with primary ACL rupture or ACL graft re-rupture.

**Technique Description:**

The patient was placed in supine position. Following examination under anesthesia, diagnostic arthroscopy was performed to assess for meniscus injuries and chondral defects. The surgical technique required 6 steps: (1) graft preparation (4-strand semitendinosus tendon, graft length was about 45 mm, grafts larger than 8 mm), (2) creating femoral socket (5-10 mm), (3) creating tibial tunnel socket (10-15 mm), (4) passing the graft, (5) tensioning the graft, and (6) graft fixation and skin closure. Patients were partial weightbearing with crutches for 3 weeks and used a hinged knee brace for 6 weeks after surgery. Return to activities was based on functional strength recovery, with a return to running targeted at 4 to 5 months after surgery.

**Results:**

All-inside ACLR with short sockets technique provides a better chance of return to preinjury level of activity with accompanied patient satisfaction as compared with anteromedial portal technique at 2 years of follow-up. This technique has favorable functional and clinical outcomes with improvement of graft maturing.

**Discussion/Conclusion:**

In conclusion, we propose that ACLR with short tibial and femoral socket technique is simple and safe with favorable clinical outcomes. This is an advantageous ACLR technique that can preserve the gracilis muscle and reduce muscle strength loss of affected limbs. In addition, the all-inside technique is circumferential filling of the socket with the graft, which might increase bone to graft contact compared with interference screws. Further study is needed to show the better clinical outcome and the earlier graft maturation compared with ordinary ACLR.

**Patient Consent Disclosure Statement:**

The author(s) attests that consent has been obtained from any patient(s) appearing in this publication. If the individual may be identifiable, the author(s) has included a statement of release or other written form of approval from the patient(s) with this submission for publication.

## Graphical abstract

**Graphical Abstract:**
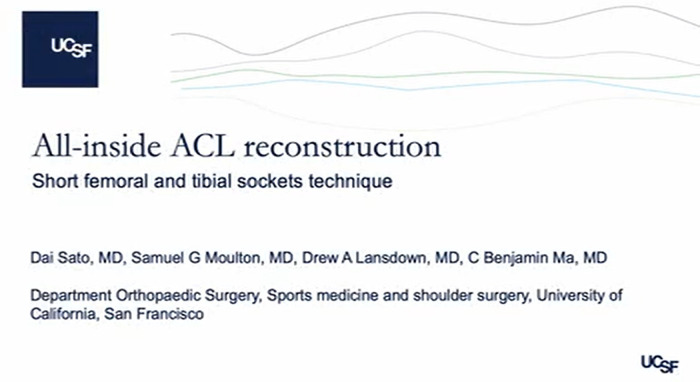
This is a visual representation of the abstract.

## Video Transcript

In this video, we demonstrate a surgical technique for all-inside anterior cruciate ligament (ACL) reconstruction (ACLR) with short femoral and tibial sockets.

The authors’ disclosures are listed.

All-inside ACLR is a novel technique that has gained attention due to its minimally invasive and graft-saving properties.^6^ However, there are no significant differences regarding patient-reported outcome scores comparing all-inside with conventional ACLR techniques.^1,5^

Regarding the maturation of an ACL graft, the contact of the graft with the bone tunnel is important.^4^ Studies have shown that graft healing starts at the periphery of the bone tunnel or socket.^2^ Therefore, we hypothesize that shortening the bone sockets during ACLR surgery can promote similar healing of the ACL graft to conventional techniques. In this video, we introduce a new technique for all-inside ACLR with short femoral and tibial sockets.

Indications for the current technique include skeletally mature individuals, older patients, those with specific activity demands, and patients who prefer the use of a hamstring allograft. It is also indicated for those who have previously undergone a bone-patellar tendon-bone autograft harvest in a revision setting. If the patient exhibits a posterior tibial slope greater than 12°, the addition of a lateral extra-articular tenodesis is recommended.

A 26-year-old female presented with left knee pain and instability. She had fallen in wet snow while skiing but did not feel a pop at the time. Later that day, while descending stairs, she felt and heard a pop, and her knee immediately swelled up.

On the examination, the patient had a normal gait with a range of motion (ROM) of 0° to 130°. She had a 2B Lachman test and a pivot shift clunk during the examination. The rest of her examination was unremarkable.

A magnetic resonance imaging confirmed the ACL tear with associated lateral tibial plateau and lateral femoral condyle bone bruises.

Given the patient's desire to return to a high-level sport including skiing, she was indicated for ACLR surgery. We will now present the key steps of the all-inside ACLR with hamstring autograft in short femoral and tibial sockets.

The semitendinosus tendon is harvested using a conventional method and then prepared for grafting. The tendon is folded over adjustable loop sutures to form a quadrupled limb construct, measuring 48 mm in length. The subsequent step involves suturing on the femoral side. The suture, using number 2 FiberWire, starts from the femoral side. It begins from the inside, passing through 2 limbs, and is then wrapped around the graft 5 to 6 times. To adjust the graft length on the tibial side, the graft's end on the tibial side is trimmed. Next, the suture is performed on the tibial side. Using number 2 FiberWire, the suture starts from the tibial side, and the graft ends are then tubularized using sutures. Beginning from the inside and passing through 2 limbs, the suture is wrapped around the graft 5 to 6 times. Finally, the graft measures 48 mm in length, with markings placed 15 mm from the tibial end and 10 mm from the femoral end.

The key steps are illustrated here:

- A quadrupled graft is prepared measuring 48 mm in length.
- Suture on the femoral side is passing through 2 limbs and is then wrapped around the graft 5 to 6 times.
- The graft's end on the tibial side is cut to adjust the length.
- Suture and tubularize on the tibial side are passed through 2 limbs and is then wrapped around the graft 5 to 6 times.
- Markings are placed 15 mm from the tibial end and 10 mm from the femoral end.

Second step is forming the femoral bone socket. First, the drill guide arm is placed into the joint and centered on the femoral ACL footprint. Small incision is made on the lateral thigh of the guide entry point down to the lateral femoral condyle. The guide sleeve is pushed down to bone and the retro reamer is brought into the joint. Retro reamer is then flipped in the femoral socket and is removed to a depth of 10 mm. After removing the retro reamer of passing sutures passed down through the guide sleeve and using the grasper, the passing sutures are retrieved from the femoral socket. Location and depth are confirmed.

Next, using the incision site for the hamstring harvest, the tibial guide is placed on the tibial cortex, while also placing the arm of the drill guide on the center of the ACL of the tibial footprint. The retro reamer is brought into the joint and flipped in the tibial socket and is reamed to adapt to 15 mm. After removing the retro reamer, nylon wire is passed through the guide sleeve are used to shuttle a passing suture through the tibia.

Graft is then loaded onto the passing suture in the femoral button sutures which are delivered out through the femoral side. Under arthroscopic visualization, the femoral suture button is delivered through the femoral socket. The button is flipped and secured on the lateral femoral condyle and visualized to ensure that it is under the iliotibial band. The graft is then delivered into the femoral socket by alternating forward pulls on the femoral side suture and pulling back on the tibial side sutures to remove slack in the system. Once the graft is docked into the femoral socket, the tibial side suspensory sutures are passed through the tibia with the passing suture and the graft is delivered into the tibial socket. The knee is then taken through an ROM to ensure proper motion and no graft impingement. A button is lowered down to the tibial suspensory sutures in tension down onto the tibia and the sutures are tied.

Postoperative radiographs demonstrate appropriate locations of the short tibial and femoral sockets.

A study indicated that the all-inside ACLR offers better prospects for returning to preinjury activity levels and improved patient satisfaction at a 2-year follow-up compared with the anteromedial portal technique.^5^ Regarding graft healing, another study suggested that the all-inside ACLR with suspensory fixation offers more favorable conditions for graft incorporation and ligamentalization within the tibial tunnel than the anteromedial portal technique with interference screws.^3^

Postoperatively, patients are expected to be partial weightbearing with crutches for 3 weeks and progress to full weightbearing and a hinged knee brace until 6 weeks after surgery. Return to activities will be based on the recovery of functional strength, with a return to running targeted at 4 to 5 months after surgery. Patients can return to sport activities 9 to 12 months after surgery.

We make sure that the graft length is not less than 45 to 55 mm at most. If the graft is shorter, the portion of tibial side graft is not placed in the tibial socket, and if the graft is longer, there is a risk of bottom out. To prevent oversize of the graft in the tibial side, the graft transection in the tibial side was stacked in the folded portion. We make sure that the drill guide is into the cortex of the lateral femur and medial tibia; if not, there is the possibility of change of bone socket length.

Advantages of the all-inside ACLR with short socket technique include circumferential filling of the socket with the graft, increasing bone and graft contact compared with interference screw fixation,^7^ less bone loss compared with conventional ACL tunnels,^9^ and preserving the gracilis muscle, potentially reducing muscle strength loss of the affected limb.^10^ Potential disadvantages include the risk of the graft bottoming out during tensioning and fixation, the graft is longer than the tibial socket,^6^ and the potential for graft micromotion with suspensory fixation.^8^

These are references.

Thank you
